# Natural Killer Immunotherapy for Minimal Residual Disease Eradication Following Allogeneic Hematopoietic Stem Cell Transplantation in Acute Myeloid Leukemia

**DOI:** 10.3390/ijms20092057

**Published:** 2019-04-26

**Authors:** Norimichi Hattori, Tsuyoshi Nakamaki

**Affiliations:** Division of Hematology, Department of Medicine, Showa University School of Medicine, Tokyo 142-8555, Japan; nakamaki@med.showa-u.ac.jp

**Keywords:** natural killer cell, immunotherapy, allogeneic hematopoietic stem cell transplantation, acute myeloid leukemia, immune checkpoint, bispecific and trispecific killer cell engagers, chimeric antigen receptors

## Abstract

The most common cause of death in patients with acute myeloid leukemia (AML) who receive allogeneic hematopoietic stem cell transplantation (allo-HSCT) is AML relapse. Therefore, additive therapies post allo-HSCT have significant potential to prevent relapse. Natural killer (NK)-cell-based immunotherapies can be incorporated into the therapeutic armamentarium for the eradication of AML cells post allo-HSCT. In recent studies, NK cell-based immunotherapies, the use of adoptive NK cells, NK cells in combination with cytokines, immune checkpoint inhibitors, bispecific and trispecific killer cell engagers, and chimeric antigen receptor-engineered NK cells have all shown antitumor activity in AML patients. In this review, we will discuss the current strategies with these NK cell-based immunotherapies as possible therapies to cure AML patients post allo-HSCT. Additionally, we will discuss various means of immune escape in order to further understand the mechanism of NK cell-based immunotherapies against AML.

## 1. Introduction

Allogeneic hematopoietic stem cell transplantation (allo-HSCT) has been recognized as the only curative therapy for patients with acute myeloid leukemia (AML). Allo-HSCT’s mode of action is primarily attributed to the graft-versus-leukemia (GVL) effect mediated by donor T-cells and natural killer (NK) cells. However, approximately 40% of the AML patients who undergo allo-HSCT will relapse, and the two-year post relapse survival among these patients is less than 20% [1,2,3,4,5,6]. With the use of targeted sequencing or flow cytometry, the persistent detection of minimal residual disease (MRD) is associated with post-transplantation relapse [7,8,9]. It is therefore important to provide additional therapies to eliminate MRD after allo-HSCT, particularly in high-risk AML. Donor lymphocyte infusion (DLI) or repeat allo-HSCT as a donor cell-based therapy has been associated with improved survival in patients who relapse after allo-HSCT [1,2,3,5,10]. Although the efficacy of therapeutic DLI in relapsed AML may be suboptimal, pre-emptive, or prophylactic, DLI may have an important role [5,11,12,13]. Use of the hypomethylating agent azacitidine appears to be effective in AML following allo-HSCT [14,15]. Additionally, pre-emptive treatment with azacitidine may prevent a relapse while monitoring for MRD (NCT01462578) [16].

**Figure 1:**
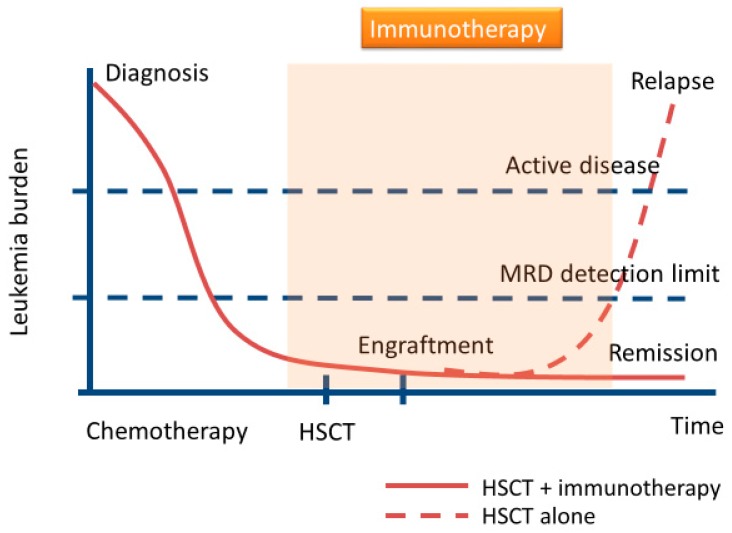
Schematic diagram of immunotherapies for minimal residual disease (MRD) eradication after allogeneic hematopoietic stem cell transplantation (allo-HSCT) in acute myeloid leukemia (AML). Some patients with AML after conventional allo-HSCT will relapse. For the prevention of relapse, immunotherapies may play an important role in the elimination of MRD.

Previously, it had been presumed that most additional therapies would not be able to suppress the proliferation of leukemia cells in the long term in relapsed AML after allo-HSCT. However, the early use of these therapies might prevent relapse in AML, and could be an important step toward improving prognosis. Recently, immunotherapies, including NK cells administration and immune checkpoint inhibitors (ICIs), have been reported as new treatment modalities after allo-HSCT in hematologic malignancies [17,18,19,20,21,22,23]. Previous studies had demonstrated that ICIs had antitumor immune responses for several solid tumors and hematologic malignancies [24,25,26]. However, their responses remained limited because of the lack of MHC classes I and II, which leads to less T-cell activation and proliferation, and is observed in ICI-resistant tumors [27,28,29]. In contrast, while NK cells express limited MHC (e.g., human leukocyte antigen (HLA)-Bw4, C1, and C2)-dependent receptors, they express non-MHC-dependent receptors including NKG2D, natural cytotoxicity receptors, CD96, T-cell immunoreceptor with Ig, and immunoreceptor tyrosine-based inhibition motif domains (TIGIT), DNAM-1, SLAMF6 (also known as NTB-A), NKRP1-B, and 2B4 [21,30,31]. Additionally, consistent with donor T-cell mediated GVL, donor T-cells’ contribution to graft versus host disease (GVHD) is dependent upon recognition of HLA disparities following allo-HSCT. While administration of some ICIs post allo-HSCT may lead to severe GVHD [17,24], donor NK cells confer alloreactivity against tumors without GVHD [32,33]. Recently, we have noted that high NK cell levels in the bone marrow microenvironment immediately following allo-HSCT were associated with better overall survival (OS) and progression-free survival [34]. Moreover, AML patients with lower TIGIT expression following allo-HSCT had superior OS and progression-free survival [35]. Therefore, strategies to activate NK cells in order to reinforce GVL effect as a pre-emptive or prophylactic immunotherapy may improve MRD clearance in high-risk AML after allo-HSCT (Figure 1). In this review, we focus on NK cell-based immunotherapies following allo-HSCT and explore emerging therapies to eradicate MRD.

## 2. Adoptive NK Cell Therapy and Cytokine-Based NK Cell Therapy

**Figure 2:**
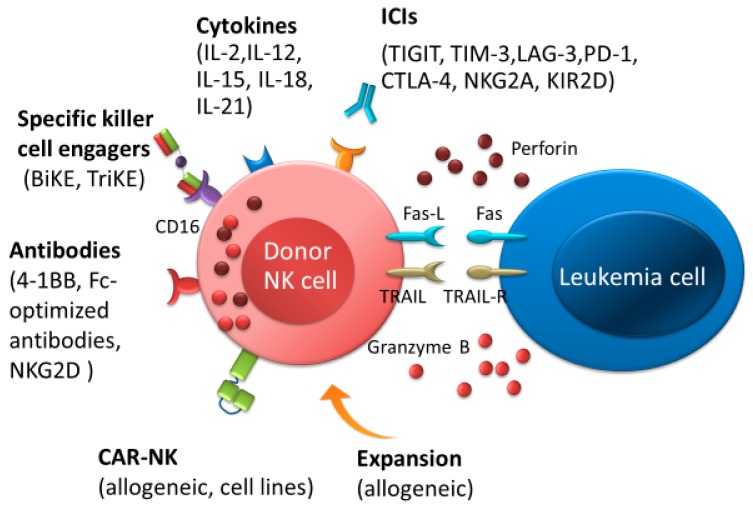
Natural killer (NK) cell immunotherapies after allo-HSCT therapeutic approaches for the elimination of AML. NK cells-based immunotherapeutic concepts are based on stimulating NK cells by cytokines or immune checkpoint inhibitors, promoting antibody-dependent T-cell-mediated cytotoxicity by antibodies or bispecific and trispecific killer cell engagers, and improving NK cell responses by adoptive transfer of NK cells, such as allogenic NK cells or chimeric antigen receptor NK cells. Abbreviations: ICIs, immune checkpoint inhibitors; BiKEs, bispecific killer cell engagers; TriKEs; trispecific killer cell engagers; CAR, chimeric antigen receptor.

**Figure 3:**
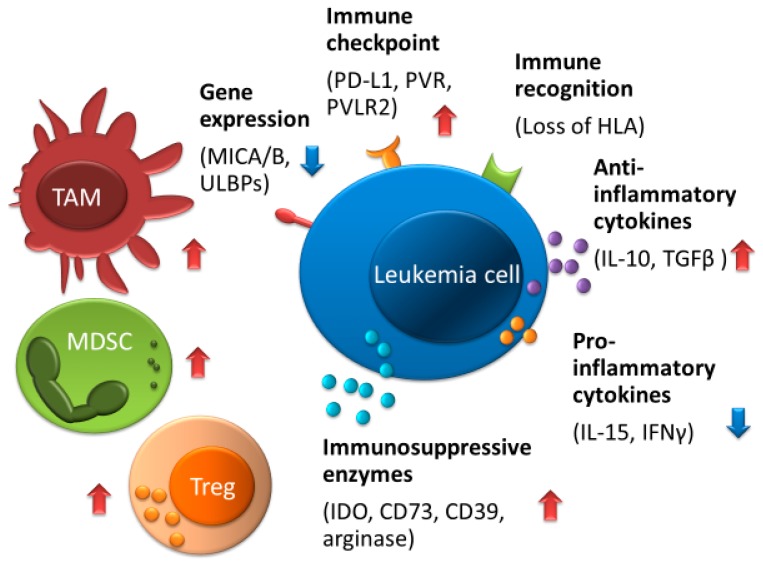
Mechanisms of immune escape against NK cells in AML. The tumor microenvironment consisted of Tregs, TAMs, and MDSCs which can interfere with the function of NK cells, and its microenvironment is increased by anti-inflammatory cytokine including TGF-β, IL-4, and IL-10 and is decreased by pro-inflammatory cytokines including IFN-γ and IL-15. Leukemia cells can produce the metabolic enzymes such as IDO, arginase, CD39, and CD73, which reduce NK cell activity. Upregulation of immune checkpoint molecules including PD-L1, PVR, and PVLR2, low expression of NKG2D ligands such as MICA/B and ULBPs, or impaired expression of HLA can contribute to evading immune surveillance by NK cells. Abbreviations: TAM, tumor-associated macrophages; MDSCs, myeloid-derived suppressor cells; Tregs, regulatory T-cells. Red arrows indicate increased expression, enzymes, cytokine production, and cell proliferation; blue arrows indicate decreased expression and cytokine production.

Previous studies have reported an association between clinical outcomes and NK cell recovery after allo-HSCT. This likely occurs because NK cells play an essential role in GVL effects and also in preventing infection following allo-HSCT [34,36,37]. To date, adoptive transfer of NK cells from allogeneic donors to patients with AML has been performed following allo-HSCT [38,39,40,41,42,43,44]. Additionally, NK cell infusion has been combined with the administration of IL-2 to boost in vivo expansion (Figure 2) [45,46,47,48]. T-regulatory cells (Tregs) are significantly increased in number following NK cell infusion and IL-2 administration, which may inhibit NK cell functionality and hinder the efficacy of adoptively transferred NK cells (Figure 3). In cases with prior IL-2-diphtheria toxin fusion protein treatment for the depletion of host Tregs, increased in vivo expansion of NK cells was noted, and relapsed/refractory AML patients were able to achieve complete remission (CR) (NCT00274846 and NCT01106950) [47]. Besides IL-2 administration, NK cells activated by IL-12, IL-15, IL-18, and IL-21 have enhanced antitumor functionality [49,50,51,52]. These cytokines also lead to an increase in varying degrees of host and/or donor CD8^+^ T-cells. Therefore, these therapies may result in adverse events, including severe GVHD. However, previous studies have demonstrated that adoptively transferred NK cells activated by these cytokines had GVL effect without life-threatening GVHD [49,50,51,52]. IL-15/IL-15Ra-Fc (ALT-803) therapy (NCT01885897), for instance, promoted an increase in CD8^+^ T-cells of the effector or effector memory phenotype without increasing Tregs, and no patient developed severe GVHD despite the induction of CD8^+^ T-cell activation [51]. One possible reason may be that the preferential expansion of NK cells mediates a reduction of GVHD by inhibiting CD8^+^ donor T-cell proliferation [53]. Although adoptive transfer of NK cells during allo-HSCT may be a promising therapy for AML, further studies are required in order to design protocols that balance the persistence of donor NK cells and host/donor T-cell activation. These studies must include the timing of transferred NK cells, NK cell dosage, combination with cytokines, the conditioning regimen, donor selection, and GVHD prophylaxis.

## 3. ICIs for Intensifying the Activation of NK Cells

**Figure 4:**
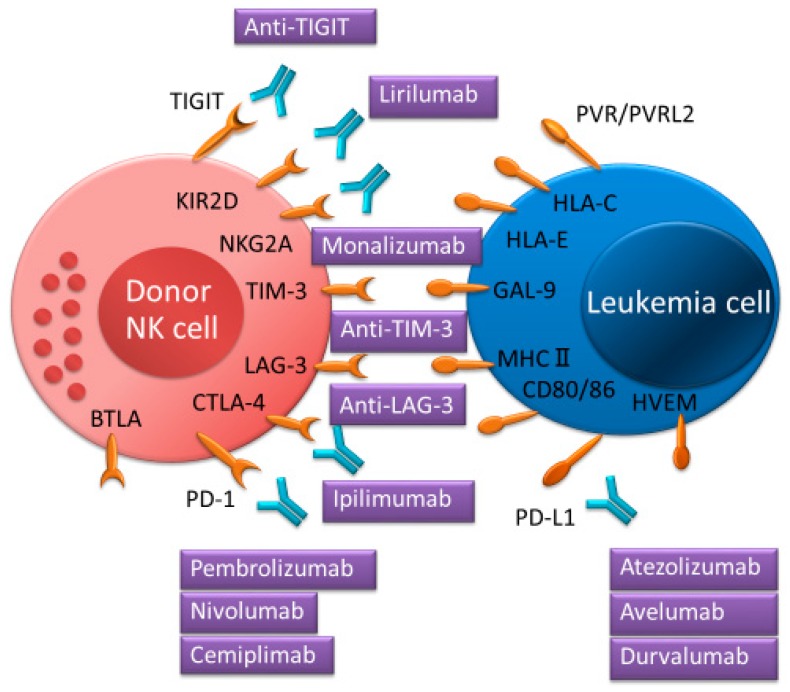
Immune checkpoint inhibitors for targeted NK cell proteins and interactions between immune checkpoint receptors and ligands enhancing NK cell function.

**Figure 5:**
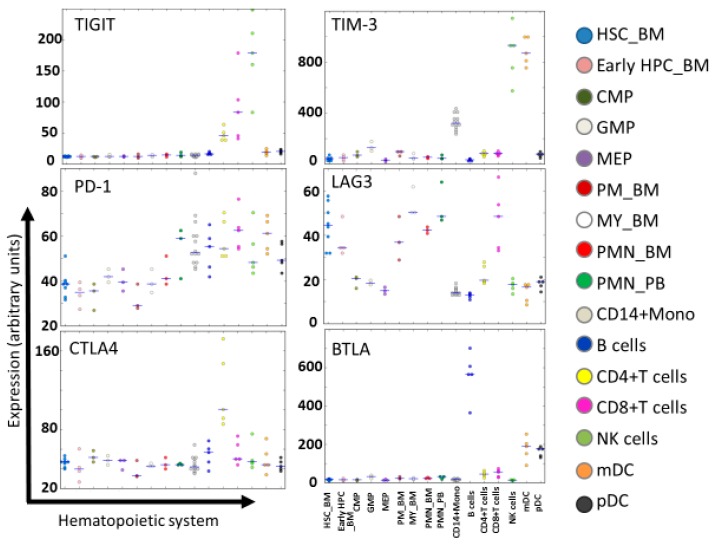
Expression of immune checkpoint receptors in normal hematopoiesis. Expression levels of TIGIT, TIM-3, PD-1, CTLA-4, LAG-3, and BTLA in the hematopoietic system at different maturation stages are shown according to HemaExplorer^54,55^, based on curated microarray data. Abbreviations: HSC_BM, hematopoietic stem cells from bone marrow; early HPC_BM, hematopoietic progenitor cells from bone marrow; CMP, common myeloid progenitor cell; GMP, granulocyte monocyte progenitors; MEP, megakaryocyte-erythroid progenitor cell; PM_BM, promyelocyte from bone marrow; MY_BM, myelocyte from bone marrow; PMN_BM, polymorphonuclear cells from bone marrow; PMN_PB, polymorphonuclear cells from peripheral blood; B-cells, CD19^+^ B-cells; NK cells; CD56^+^ natural killer cells; mDC, CD11c^+^ myeloid dendritic cells; pDC, CD123^+^ plasmacytoid dendritic cells.

NK cells express various co-inhibitory receptors, including killer immunoglobulin-like receptors (KIRs), NKG2A, programmed cell death protein 1 (PD-1), cytotoxic T-lymphocyte-associated protein 4 (CTLA-4), T-cell immunoglobulin, and mucin domain-containing protein 3 (TIM-3), TIGIT, and lymphocyte activation gene 3 (LAG-3). These receptors have recently been recognized as immune checkpoints (Figure 2 and Figure 4). T-cells also express these immune checkpoints in which PD-1, CTLA-4, LAG-3, and B- and T-lymphocyte attenuator (BTLA) had greater expression than in NK cells (Figure 5) [54,55]. In order to block a receptor’s inhibitory signal, NK cell activation and leukemia cell killing are induced by a cognate ligand by ICIs. Therefore, administration of ICIs in the first few months post allo-HSCT might be a powerful tool for eradicating MRD following AML. Several clinical trials for ICIs as monotherapy or as part of combination treatment for AML after allo-HSCT have been reported recently [17,20,24,56,57,58]. CTLA-4 (e.g., ipilimumab) and PD-1 (e.g., nivolumab) blockade have been administered following allo-HSCT in several hematologic malignancies, and beneficial GVL responses have been achieved. However, serious immune-related adverse events and/or severe GVHD were accompanied by exposure to these two ICIs [59]. This might explain how increased donor-derived alloreactive T-cells might cause life-threatening GVHD [57,58]. It would be interesting to test whether NK cell-specific ICIs might enhance anti-leukemia activity without aggravating GVHD more than CTLA-4 or PD-1 blockade.

NK cells express various immune checkpoint receptors such as KIR-2D, NKG2A, PD-1, CTLA-4, TIGIT, TIM-3, LAG-3, and BTLA, which can interact with their cognate ligands on tumor cells or on several immune cells. Immune checkpoint inhibitors can interrupt their receptor’s inhibitory signal.

IPH2101, also known as anti-KIR1-7F9 monoclonal antibody (mAb; lirilumab is a recombinant version of this mAb), blocks common inhibitory KIRs (KIR2DL/DS-1, -2, and -3), which bind to HLA-C alleles and augment NK cell-mediated killing in HLA-C-expressing leukemia cells [60]. Although this KIR2D blockade showed no clinical effectiveness [61] in a phase 2 trial in smoldering multiple myeloma (NCT01248455), adoptive transfer of NK cells combined with IPH2101 after allo-HSCT may have a therapeutic benefit. With lirilumab as with ICIs, CD94-NKG2A receptors on NK cells primarily recognize HLA-E, which is expressed by leukemia cells. Anti-NKG2A mAb (monalizumab) administration showed anti-leukemia effects in hematologic malignancies [62,63,64]. Previous studies have demonstrated that anti-NKG2A mAb can induce NKG2A^+^ NK cell killing activity against HLA-E-expressing leukemia cells in vitro and in vivo [62,63,64]. Additionally, reduced numbers of NKG2A^+^ NK cells after allo-HSCT are associated with the occurrence of severe GVHD [65,66]. Moreover, NKG2A^+^ NK cells inhibited T-cell proliferation and activation and might prevent GVHD [66]. Therefore, NKG2A^+^ NK cells may play a crucial role in GVHD and GVL effect following allo-HSCT, and monalizumab administration may have a promising clinical role following allo-HSCT.

TIGIT is expressed by both T and NK cells, and its ligands are the poliovirus receptors PVR (also known as CD155) and PVRL2 (also known as CD112) [30]. PVR is overexpressed in several tumors including AML, and its overexpression has been linked to a poor prognosis in AML [35,67,68]. Meanwhile, AML patients with higher TIGIT expression after allo-HSCT had an inferior prognosis [35]. Previous studies have shown that blockade of TIGIT could prevent the exhaustion of NK cells and enhance NK cell-dependent antitumor effect [20,69]. Additionally, CMV-induced adaptive NK cells had less TIGIT expression compared to conventional NK cells, and overcame myeloid-derived suppressor cell (MDSC)-mediated immune suppression. The cytotoxic function of NK cells co-cultured with MDSCs against tumor cells could be restored by blockade of TIGIT [70]. Unlike PD-1 and CTLA-4 inhibitors, NK cells may play a critical role in TIGIT-based immunotherapy, and blockade of TIGIT may have therapeutic effects in GVL by controlling NK-cell activity after allo-HSCT. However, because blockade of TIGIT can also promote T-cell activity [71], it is imperative to optimize the clinical setting to prevent severe GVHD and intensify the GVL effect in AML patients undergoing allo-HSCT.

TIM-3 is expressed on all mature CD56^dim^CD16^+^ NK cells and activated immature CD56^bright^CD16^−^ NK cells. Its ligand is galectin-9 [72], which induces interferon-gamma (IFN-γ) production by NK cells [73]. TIM-3 blockade restores NK cell exhaustion and leads to an increased NK cell cytotoxicity in several cancers [74,75], whereas the TIM-3 antibody agonist leads to a decrease in the cytotoxicity of NK cells [72]. TIM-3 blockade reduces NK cell-mediated killing of pancreatic cancer cell lines [76]. Although a phase 1 study evaluating the blockade of TIM-3 (TSR-022) in advanced solid tumors is in progress (NCT02817633), further studies will be needed to determine the precise role of TIM-3 in AML after allo-HSCT.

LAG-3 (also known as CD223) is a ligand which has been identified as MHC class II. It is widely expressed not only on activated T and NK cells but also on dendritic and B-cells (Figure 5) [75,77,78]. LAG-3 is involved in inhibiting T-cell effector function, and blockade of LAG-3 promotes T-cell proliferation in vitro [79]. However, the function of LAG-3 on NK cells remains unclear. Blockade of LAG-3 had no effect on NK-cell-mediated cytotoxicity [80]. Further investigation on the role of LAG-3 on NK cells is necessary.

BTLA (also known as CD272), which belongs to the immunoglobulin superfamily, is expressed by most lymphocytes (Figure 5). BTLA acts as a negative modulator of immune responses regulating T-cell activation and proliferation [77,81,82]. Its ligand, herpesvirus entry mediator (HVEM, also known as TNFSF14), is expressed in several tumor cells [82,83]. Blocking BTLA-HVEM interaction leads to a decrease in suppressor T-cells in the tumor microenvironment and enhances antitumor immunity [84]. Additionally, BTLA blockade promotes an increase in NKT-cells and expression of cytotoxic marker genes [85]. However, the functional role of BTLA on NK cells is controversial and requires further investigation.

## 4. Bi/Trispecific Engagers and Chimeric Antigen Receptors (CAR) NK Cells

Bispecific and trispecific killer cell engagers (BiKEs and TriKEs), which are composed of a single-chain variable fragment (scFv) containing a variable heavy and variable light chain of an antibody, can specifically target both CD16 expressed on NK cells and tumor antigens (Figure 2). Previous studies have shown that NK-cell-mediated cytotoxicity could occur by CD16 × CD33 (1633) BiKEs that ligated CD16 on NK cells and CD33 on tumor cells, including myelodysplastic syndromes (MDS) and AML [86,87]. Recently, 161533 TriKE, which is an NK-cell stimulatory cytokine with IL-15 added onto BiKE, has been found to restore NK cell proliferation and function through a low expression of TIGIT in NK cells. It is also able to enhance NK-cell-mediated cytotoxicity against MDS cells more than 1633 BiKE [88]. Moreover, NK cells treated with 161533 TriKE can overcome immune suppression mediated by MDSCs. Although IL-15 also stimulates cytotoxic T-cells, 161533 TriKE induces the proliferation of NK cells with minimal effect on T-cells [87]. Therefore, the administration of this agent after allo-HSCT may be a potentially promising treatment to decrease relapse of AML after allo-HSCT with less T-cell-mediated GVHD.

Chimeric antigen receptors (CARs) consist of scFv (extracellular domains) combined with CD3ζ, DAP10, or DAP12 as intracellular signal domains, and CD28, 4-1BB (also known as CD137), and 2B4 (also known as CD244) as costimulatory domains (Figure 2) [89,90,91,92,93]. In a murine allogeneic transplant model using donor-derived CD19-CAR T-cells, allogeneic CAR T-cells eliminated acute lymphoblastic leukemia [94]. However, its administration caused lethal GVHD. Additionally, CD123-redirected T-cells (CART123) eliminated AML and also eradicated normal hematopoietic stem cells (HSCs) in a mouse model because CD123 is highly expressed in HSC [95]. In a phase 1 clinical trial of CD33-CAR NK cells for relapsed and refractory AML patients (NCT02944162), the administration of CD33-CAR NK cells was not clinically efficacious [92]. Recently, cord blood-derived NK cells with CAR-CD19, IL-15, and inducible caspase-9-based suicide gene (iC9) (iC9/CAR.19/IL15-transduced CB-NK cells) enhanced their cytotoxicity against CD19-expressing tumors in a murine model [96]. CAR NK cells may provide a cost-effective treatment with a reduced risk of GVHD compared to CAR T-cells, but further clinical studies will be needed to demonstrate the safety and efficacy of CAR NK cells against AML following allo-HSCT.

## 5. AML Survival Mechanism against NK Cells

NK cell-based immunotherapies may emerge as a promising option for elimination of AML following allo-HSCT, but several factors may limit NK cell-based immunotherapies (Figure 3) [97,98,99]. For instance, the tumor microenvironment, which includes Tregs, tumor-associated macrophages, and MDSCs, which interfere with the function of NK cells, is a major limitation to the effectiveness of NK cells [70,87,100,101]. In addition, the tumor microenvironment possesses increased anti-inflammatory cytokines, such as TGF-β, IL-4, and IL-10, which cause immune evasion and result in decreased pro-inflammatory cytokines, including IFN-γ and IL-15, which stimulate NK cell activation [51,99,102]. Moreover, leukemia cells produce several enzymes such as indoleamine 2,3-dioxygenase-1, arginase, prostaglandin-E2, CD39, and CD73, which reduce NK-cell proliferation and/or activity [98,99,100,103,104,105,106].

The incidence of HLA loss following allo-HCT is one of the major immune escape mechanisms that lead to relapse in AML, and may account for approximately one third of all relapses [107]. Because loss of mismatched HLA through copy-neutral loss of heterozygosity results in the elimination of the incompatible HLA alleles while keeping the expression of HLA class I molecules, cytotoxic killing by NK cells does not occur. Also, recent studies have shown that the downregulation of HLA class II molecules (HLA-DPA1, HLA-DPB1, HLA-DQB1, and HLA-DRB1) and their related molecules (CIITA, IFI30, HLA-DMA, HLA-DMB, and CD74) could allow leukemia relapse after allo-HSCT [108].

Patients who exhibited a high expression of CD200, CD47, PD-L1, PVR, or PVRL2, which is associated with an immune response or immune checkpoints, had a poor prognosis [35,67,109,110,111]. In addition to PD-1, exhausted T-cells, including exhausted CD8^+^ T-cells, express inhibitory receptors such as CTLA-4, LAG-3, and TIM-3 [21,112,113]. T-cell exhaustion contributes to AML relapse after allo-HSCT [113]. In contrast to T-cell exhaustion, expression of activating NKG2D ligands such as MHC class I-related chain A (MICA) and UL16-binding protein 1 (ULBP1) on AML cells at diagnosis is associated with an improved OS and a reduced incidence of relapse [114]. Activated NKG2D on NK cells recognizes NKG2D ligands (MICA/B and ULBPs) and enables the induction of NK-cell-mediated cytotoxicity on AML cells [115,116]. AML cells which express low levels of NKG2D ligands are able to evade immune surveillance by NK cells [115].

Janus kinase (JAK) mutations affect the interferon (IFN) signaling pathway by inducing an increase in STAT1 expression, the loss of beta-2-microglobulin, which can detect HLA class I antigen processing, and the loss of PTEN, which increases the production of immunosuppressive cytokines such as VEGF and can increase STAT3 expression. These mutations, which represent various mechanisms of resistance to ICIs, have been reported in several cancers [117,118,119,120], but it remains unknown in the case of AML. These studies demonstrated the association between T-cell activity and resistance to ICIs. However, the mechanisms of resistance to ICIs on NK cells are less well explored and require further elucidation.

## 6. Conclusions

Currently, there are numerous NK cell-based immunotherapies for AML post allo-HSCT that have been incorporated into pre-clinical and clinical trials. We have described some clinical trials associated with NK cell-based immunotherapies (Table 1). NK cell immunotherapies such as adoptive NK cells, cytokine-based therapies, ICIs, and bi/trispecific engagers have the potential to significantly enhance conventional therapies for the elimination of AML after allo-HSCT. In the future, combinations of these approaches require to be optimized to further enforce donor NK-cell mediated GVL in AML patients who received allo-HSCT. Moreover, for the next generation of NK-cell immunotherapies, therapeutic approaches based on CAR-engineered NK cells, memory-like NK cells, NKT-cells, and induced pluripotent stem cell-derived NK cells may be considered in a future study [49,89,121,122,123,124]. However, particularly with the use of ICIs, especially PD-1 or CTLA-4 blockade, after allo-HSCT, there is a distinct need for caution due to the risk of GVHD-related mortality. These ICIs appear to promote T-cell activity more than NK-cell activity against leukemia cells. Well-designed clinical trials should be required to demonstrate the safety and efficacy of these therapies. In addition, because NK cells have a short lifespan compared to T-cells, further improvements in manufacturing and expansion techniques are needed. Previously, immunotherapies had primarily focused on T-cell-mediated cytotoxicity. Thus, some mechanisms for NK-cell immunotherapies, including immune escape of AML, remain unclear. Further studies will be needed to predict which type of AML after allo-HSCT will be affected by NK-cell immunotherapies. The collection of large patient series and datasets will allow the investigation of the various factors which may potentially influence NK-cell immune responses. These factors will include the expression genes, mutations, alterations of resistance to immunotherapies in leukemia cells, tumor microenvironment consisting of Tregs, tumor-associated macrophages, MDSCs, and the production of cytokines.

## Figures and Tables

**Table 1:** Selected list of clinical trials in NK cell-based immunotherapies.

**Adoptive NK Cells**						
	**Clinical Trial**	**Cytokines**	**Disease**	**Phase**	**Status**	**allo-HSCT**
	NCT02229266	IL-2	AML	II	Recruiting	
	NCT00394381	IL-2	AML, MDS	I/II	Completed	yes
	NCT01370213	IL-2	AML, MDS	II	Unknown	yes
	NCT01947322	IL-2	AML	I/II	Completed	yes
	NCT03068819	IL12, IL15, IL18	AML	I	Recruiting	yes
	NCT02782546	ALT-803 (IL-15)	AML	II	Recruiting	yes
	NCT02890758	ALT-803 (IL-15)	HMs, solid tumors	I	Recruiting	
	NCT00460694	IL-2	HMs	I/II	Completed	yes
	NCT01823198	IL-2	HMs	I/II	Recruiting	yes
	NCT02809092	IL-21	AML	I/II	Recruiting	
	NCT03300492	-	AML, MDS	I/II	Recruiting	yes
**Immune Checkpoint Inhibitors**						
	**Clinical Trial**	**Target**	**Disease**	**Phase**	**Status**	**allo-HSCT**
IPH2101						
	NCT01256073	KIR	AML	I	Completed	
IPH2102						
	NCT01687387	KIR	AML	II	Completed	
Lirilumab						
	NCT01687387	KIR2D	AML	II	Completed	
	NCT02399917	KIR2D	AML	II	Completed	
Monalizumab						
	NCT02921685	NKG2A	HMs	I	Recruiting	yes
Nivolumab						
	NCT03600155	PD-1 and CTLA-4	AML	I	Recruiting	yes
	NCT02846376	PD-1 and/or CTLA-4	AML, MDS	I	Recruiting	yes
	NCT01822509	PD-1 or CTLA-4	HMs	I	Active, not recruiting	yes
Pembrolizumab						
	NCT02981914	PD-1	AML, MDS, ML	I	Recruiting	yes
Atezolizumab						
	NCT02862275	PD-L1	HMs, solid tumors	I	Recruiting	
Avelumab						
	NCT02953561	PD-L1	AML	I/II	Recruiting	
Durvalumab						
	NCT02775903	PD-L1	AML, MDS	II	Active, not recruiting	
Ipilimumab						
	NCT03912064	CTLA-4	AML, MDS	I	Not yet recruiting	yes
	NCT00060372	CTLA-4	AML, solid tumors	I	Completed	yes
OMP-313M32						
	NCT03119428	TIGIT	solid tumors	I	Active, not recruiting	
MTIG7192A						
	NCT03563716	TIGIT and PD-L1	solid tumors	II	Active, not recruiting	
AB154						
	NCT03628677	TIGIT	solid tumors	I	Recruiting	
TSR-022						
	NCT02817633	TIM-3 and PD-1	solid tumors	II	Not yet recruiting	
	NCT02817633	TIM-3	solid tumors	I	Recruiting	
MBG453						
	NCT03066648	TIM-3	AML, MDS	I	Recruiting	
BMS-986016/BMS-936558						
	NCT02061761	LAG-3	ML	I/II	Recruiting	
Sym022						
	NCT03489369	LAG-3	ML, solid tumors	I	Recruiting	
**NK Cell Engagers**						
	**Clinical Trial**	**Target**	**Disease**	**Phase**	**Status**	
TriKEs						
	NCT03214666	CD16/IL-15/CD33	AML, MDS	I/II	Not yet recruiting	
**CAR-NK Cells**						
	**Clinical Trial**	**Target**	**Disease**	**Phase**	**Status**	**Origin of NK Cells**
	NCT02742727	CD7	AML, ALL, ML	I/II	Unknown	NK-92
	NCT02944162	CD33	AML	I/II	Unknown	NK-92
	NCT03579927	CD19	ML	I/II	Not yet recruiting	UCB
	NCT03056339	CD19	ALL, ML	I/II	Recruiting	UCB
	NCT02892695	CD19	ALL, ML	I/II	Recruiting	NK-92
	NCT01974479	CD19	ALL	I	Suspended	Haploidentical donor NK cells
	NCT00995137	CD19	ALL	I	Completed	Expanded donor NK cells

Abbreviations: allo-HSCT, allogeneic hematopoietic stem cell transplantation; AML, acute myeloid leukemia; MDS, myelodysplastic syndromes; HMs, hematological malignancies; ML, malignant lymphoma; ALL, acute lymphoblastic leukemia.

## Abbreviations

allo-HSCT	allogeneic hematopoietic stem cell transplantation
AML	acute myeloid leukemia
GVL	graft-versus-leukemia
NK	Natural killer
MRD	minimal residual disease
DLI	Donor lymphocyte infusion
ICIs	immune checkpoint inhibitors
HLA	human leukocyte antigen
TIGIT	T-cell immunoreceptor with Ig and immunoreceptor tyrosine-based inhibition motif domains
GVHD	graft versus host disease
OS	overall survival
Tregs	T-regulatory cells
CR	complete remission
KIRs	killer immunoglobulin-like receptors
PD-1	programmed cell death protein 1
CTLA-4	cytotoxic T-lymphocyte-associated protein 4
TIM-3	T-cell immunoglobulin and mucin domain-containing protein 3
LAG-3	lymphocyte activation gene 3
BTLA	B- and T-lymphocyte attenuator
MDSC	myeloid-derived suppressor cell
HVEM	herpesvirus entry mediator
BiKEs	bispecific killer cell engagers
TriKEs	trispecific killer cell engagers
MDS	myelodysplastic syndromes
CARs	chimeric antigen receptors
HSCs	hematopoietic stem cells
MICA	MHC class I-related chain A
ULBP1	UL16-binding protein 1
HSC_BM	hematopoietic stem cells from bone marrow
early HPC_BM	hematopoietic progenitor cells from bone marrow
CMP	common myeloid progenitor cell
GMP	granulocyte monocyte progenitors
MEP	megakaryocyte-erythroid progenitor cell
PM_BM	promyelocyte from bone marrow
MY_BM	myelocyte from bone marrow
PMN_BM	polymorphonuclear cells from bone marrow
PMN_PB	polymorphonuclear cells from peripheral blood
B-cells	CD19^+^ B-cells
NK cells	CD56^+^ natural killer cells
mDC	CD11c^+^ myeloid dendritic cells
pDC	CD123^+^ plasmacytoid dendritic cells
HMs	hematological malignancies
ML	malignant lymphoma
ALL	acute lymphoblastic leukemia
JAK	Janus kinase
IFN	interferon
